# Fibrin-targeting molecular MRI in inflammatory CNS disorders

**DOI:** 10.1007/s00259-022-05807-8

**Published:** 2022-05-04

**Authors:** Johannes Lohmeier, Rafaela V. Silva, Anna Tietze, Matthias Taupitz, Takaaki Kaneko, Harald Prüss, Friedemann Paul, Carmen Infante-Duarte, Bernd Hamm, Peter Caravan, Marcus R. Makowski

**Affiliations:** 1 Department of Radiology, Charité - Universitätsmedizin Berlin, corporate member of Freie Universität Berlin and Humboldt Universität zu Berlin, Campus Charité Mitte (CCM), Charitéplatz 1, 10117 Berlin, Germany; 2 Experimental and Clinical Research Center, a cooperation between the Max Delbrück Center for Molecular Medicine in the Helmholtz Association and Charité Universitätsmedizin Berlin, Berlin, Germany; 3 Charité – Universitätsmedizin Berlin, corporate member of Freie Universität Berlin and Humboldt-Universität zu Berlin, Einstein Center for Neurosciences Berlin, Charitéplatz 1, 10117 Berlin, Germany; 4 Institute of Neuroradiology, Charité - Universitätsmedizin Berlin, corporate member of Freie Universität Berlin and Humboldt Universität zu Berlin, Campus Charité Mitte (CCM), Charitéplatz 1, 10117 Berlin, Germany; 5 Center for the Evolutionary Origins of Human Behavior, Kyoto University, Inuyama, Aichi, 484-8506 Japan; 6 Department of Neurology and Experimental Neurology, Charité - Universitätsmedizin Berlin, corporate member of Freie Universität Berlin and Humboldt Universität zu Berlin, Campus Charité Mitte (CCM) and German Center for Neurodegenerative Diseases (DZNE) Berlin, Charitéplatz 1, 10117 Berlin, Germany; 7 NeuroCure Clinical Research Center and Experimental and Clinical Research Center, Charité - Universitätsmedizin Berlin, corporate member of Freie Universität Berlin and Humboldt Universität zu Berlin, Berlin, Germany; 8 Max Delbrueck Center for Molecular Medicine in the Helmholtz Association (MDC), Lindenberger Weg 80, 13125 Berlin, Germany; 9 A. A. Martinos Center for Biomedical Imaging, Institute for Innovation in Imaging, Massachusetts General Hospital, Harvard Medical School, 149 Thirteenth Street, Suite 2301, Charlestown, MB 02129 USA; 10 Department of Radiology, Klinikum Rechts der Isar, Technische Universität München, Ismaninger Str. 22, 81675 München, Germany

**Keywords:** Neuroinflammation, Multiple sclerosis, Molecular MRI, EAE, Fibrin

## Abstract

**Background:**

Fibrin deposition is a fundamental pathophysiological event in the inflammatory component of various CNS disorders, such as multiple sclerosis (MS) and Alzheimer’s disease. Beyond its traditional role in coagulation, fibrin elicits immunoinflammatory changes with oxidative stress response and activation of CNS-resident/peripheral immune cells contributing to CNS injury.

**Purpose:**

To investigate if CNS fibrin deposition can be determined using molecular MRI, and to assess its capacity as a non-invasive imaging biomarker that corresponds to inflammatory response and barrier impairment.

**Materials and methods:**

Specificity and efficacy of a peptide-conjugated Gd-based molecular MRI probe (EP2104-R) to visualise and quantify CNS fibrin deposition were evaluated. Probe efficacy to specifically target CNS fibrin deposition in murine adoptive-transfer experimental autoimmune encephalomyelitis (EAE), a pre-clinical model for MS (*n* = 12), was assessed. Findings were validated using immunohistochemistry and laser ablation inductively coupled plasma mass spectrometry. Deposition of fibrin in neuroinflammatory conditions was investigated and its diagnostic capacity for disease staging and monitoring as well as quantification of immunoinflammatory response was determined. Results were compared using *t*-tests (two groups) or one-way ANOVA with multiple comparisons test. Linear regression was used to model the relationship between variables.

**Results:**

For the first time (to our knowledge), CNS fibrin deposition was visualised and quantified in vivo using molecular imaging. Signal enhancement was apparent in EAE lesions even 12-h after administration of EP2104-R due to targeted binding (*M* ± *SD*, 1.07 ± 0.10 (baseline) vs. 0.73 ± 0.09 (EP2104-R), *p* = .008), which could be inhibited with an MRI-silent analogue (*M* ± *SD*, 0.60 ± 0.14 (EP2104-R) vs. 0.96 ± 0.13 (EP2104-La), *p* = .006). CNS fibrin deposition corresponded to immunoinflammatory activity (R^2^ = 0.85, *p* < .001) and disability (R^2^ = 0.81, *p* < .001) in a model for MS, which suggests a clinical role for staging and monitoring. Additionally, EP2104-R showed substantially higher SNR (*M* ± *SD*, 6.6 ± 1 (EP2104-R) vs. 2.7 ± 0.4 (gadobutrol), *p* = .004) than clinically used contrast media, which increases sensitivity for lesion detection.

**Conclusions:**

Molecular imaging of CNS fibrin deposition provides an imaging biomarker for inflammatory CNS pathology, which corresponds to pathophysiological ECM remodelling and disease activity, and yields high signal-to-noise ratio, which can improve diagnostic neuroimaging across several neurological diseases with variable degrees of barrier impairment.

**Supplementary Information:**

The online version contains supplementary material available at 10.1007/s00259-022-05807-8.

## Introduction

**Fig. 1:**
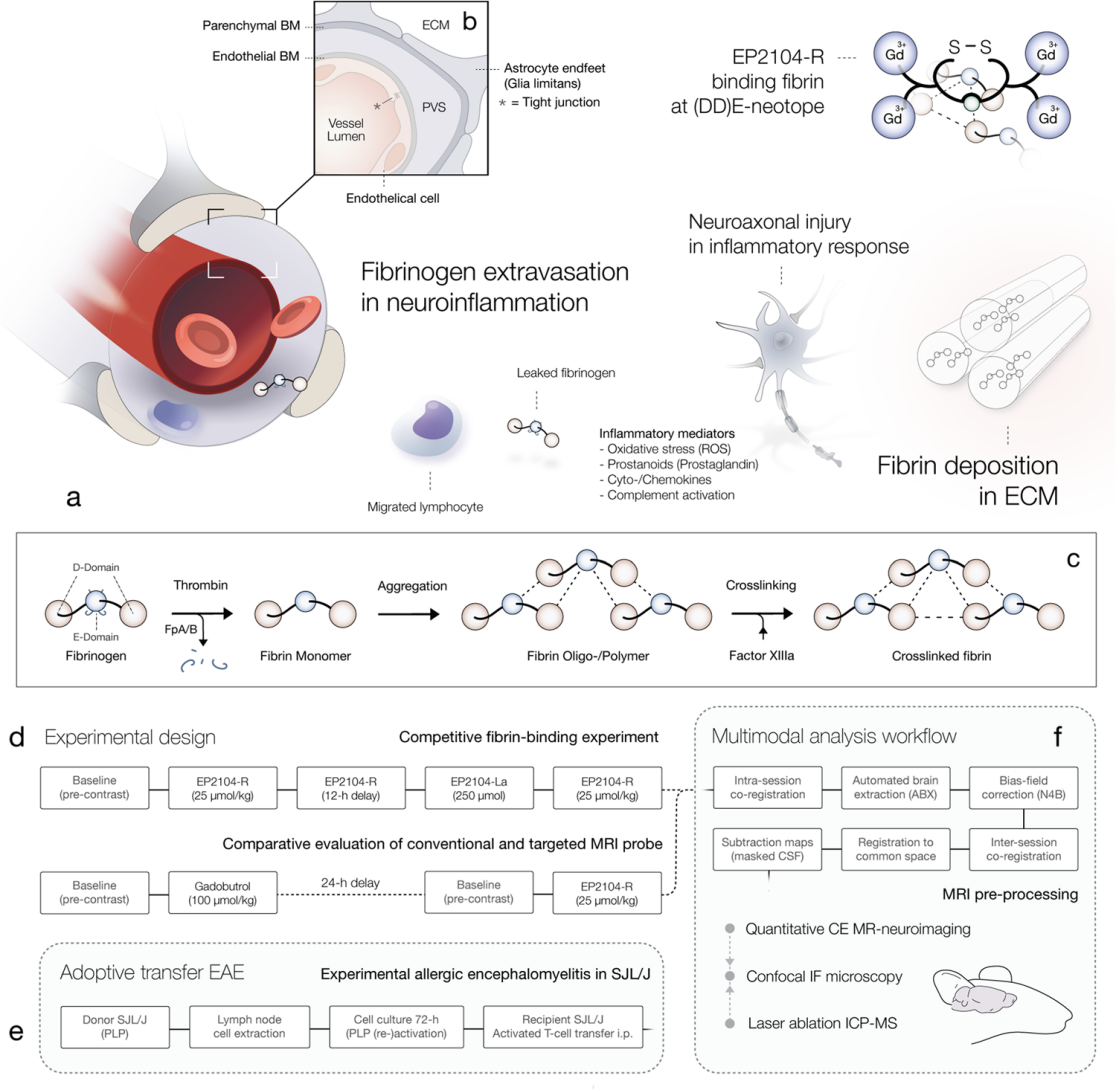
Fibrin deposition in inflammatory CNS disorders. Dysfunction of the BBB is a hallmark of acute CNS inflammation and one of the earliest events in the pathophysiology of several CNS disorders. Increased barrier permeability is accompanied by extravasation of plasma components and small externally administered compounds into the interstitial space (see **a**) and results in fibrin(ogen) deposition in the perivascular space (PVS) and ECM, which promotes inflammatory response and immune migration into the CNS. Panel **b** demonstrates a cross section of the neurovascular unit, which depicts the ensemble of tight junctions, endothelial cells, basement membranes (BM) and astrocytic endfeet that implements the barrier function. Panel **c** shows the conversion of fibrinogen to fibrin within the coagulation pathway. In the inflammatory component of various CNS diseases, such as MS, aberrant or auto-reactive leucocytes migrate into the CNS. Migrated immune cells promote further CNS migration of peripheral monocytes/macrophages, which accumulate in the PVS and eventually migrate into the interstitial space. Activated peripheral and resident immune cells form clusters around CNS entry points and induce neuroaxonal injury. EP2104-R, a peptide-conjugated Gd-based (Gd^3+^) positive contrast agent, binds fibrin with high affinity, which is visualised and quantified using molecular MRI. Panel **d** demonstrates experimental designs for the competitive fibrin binding assay and comparative evaluation of EP2104-R and a clinically used (non-targeted) MRI contrast agent. Panel **e** illustrates the induction of murine adoptive-transfer EAE model, a common model for MS. Epitope-primed and (re-)activated T-cells from SJL/J donor mice, immunised with PLP, were transferred to naïve syngeneic recipients, which rapidly develop a characteristic inflammatory demyelinating CNS disorder. Last panel (**f**) illustrates the multimodal analysis workflow for validation of imaging findings from quantitative (contrast-enhanced) MRI with immunofluorescence confocal microscopy (IF-CM) and laser ablation inductively coupled plasma mass spectrometry (LA-ICP MS). BM, basement membrane; ECM, extracellular matrix; PVS, perivascular space; ROS, reactive oxygen species; FpA/B, fibrinopeptide A/B; CSF, cerebrospinal fluid; PLP, proteolipid

The entire central nervous system (CNS) is in a highly privileged state due to physiological barriers that strictly regulate interaction with circulating blood components. Dysfunction of these barriers is a hallmark of acute CNS inflammation, which results in leakage of plasma components, such as fibrinogen, immunoglobulins or albumin, into the interstitial space. Fibrinogen (340 kDa) is an important effector protein within the coagulation pathway and innate immune response [1]. Synthesised by hepatocytes as an acute phase reactant, the plasma concentration of fibrinogen varies dependent on the state of inflammation. Upon activation of the coagulation pathway, fibrinogen is converted to the soluble fibrin monomer (see Fig. 1), which self-assembles to an insoluble mesh network that occludes further blood passage. Beyond its traditional role in coagulation, fibrin(ogen) is an active participant in neuroinflammatory processes through ligand-receptor binding mechanisms that activate downstream signalling pathways which mediate neuroinflammation and neurodegeneration [1] and, thereby, contribute to the pathophysiology of several neurological diseases, including Alzheimer’s disease [2] and multiple sclerosis (MS) [3, 4]. Disruption of the barrier function is one of the earliest events in MS, resulting in CNS fibrin(ogen) deposition, which was shown to inhibit the regeneration of myelin and promote neuroinflammation, glial scar formation, peripheral macrophage infiltration and microglial response (see [1] for a detailed review). The activation of CNS-resident microglia and peripheral macrophages [5, 6] in addition to the induction of an oxidative stress response [7] was identified as important key mechanism behind these proinflammatory effects.

MS is the most common immune-mediated neuroinflammatory and neurodegenerative disorder of the CNS and a major cause of disability in early adulthood in the Northern Hemisphere [8]. MRI plays a central role in MS patient management; however, clinical imaging is known to suffer from poor specificity for demyelinating lesions as well as low sensitivity for cortical lesions, which is a main cause of long-term disability and cognitive impairment [9]. Due to the weak association between imaging and the degree of disability in patients—which remains a relevant issue to date [10, 11]—there is a need for the development of novel neuroimaging approaches that provide better agreement between clinical and imaging measures, while potentially preparing ground for more predictive and individualised therapeutic frameworks.

Molecular imaging is known to provide biologically relevant information regarding pathophysiological remodelling [12, 13]. Due to increased barrier permeability in acute CNS inflammation, small externally administered compounds, such as targeted contrast media, may pass the blood–brain barrier (BBB) and bind target structures in the extracellular matrix (ECM). EP2104-R is an intermediate molecular-weight MRI probe (< 5 kDa) comprising a short, disulfide bridged cyclic peptide conjugated to 4 Gd-DOTA chelates, which results in 18-fold higher relaxivity (MR signal potency) than Gd-DOTA itself upon fibrin binding [14]. Its cyclic peptide confers high-affinity binding to a highly conserved neotope within fibrin’s (DD)E complex [15]. EP2104-R binds to fibrin in several species including human primates (1.7 ± 0.5 µM) and rodents, but does not bind to fibrinogen or serum proteins [14]. Up to the present, (pre-)clinical assessment was primarily focused on cardiovascular imaging [12], but EP2104-R was also found to be useful for the imaging of liver inflammation [16] and lung fibrosis [17]. Based on excellent biocompatibility, the probe was advanced into phase II clinical trials investigating thrombosis imaging [18], where no major adverse effects were reported.

In summary, we hypothesised that fibrin deposition in inflammatory CNS disorders might be determined using molecular MRI (see Fig. 1). We investigated the efficacy and specificity of fibrin-targeting MRI and tested our hypothesis in a murine model for MS, where we showed that fibrin deposition corresponds to underlying tissue damage and immunoinflammatory activity.

## Materials and methods

### In vitro* fibrin binding assay*

**Fig. 2:**
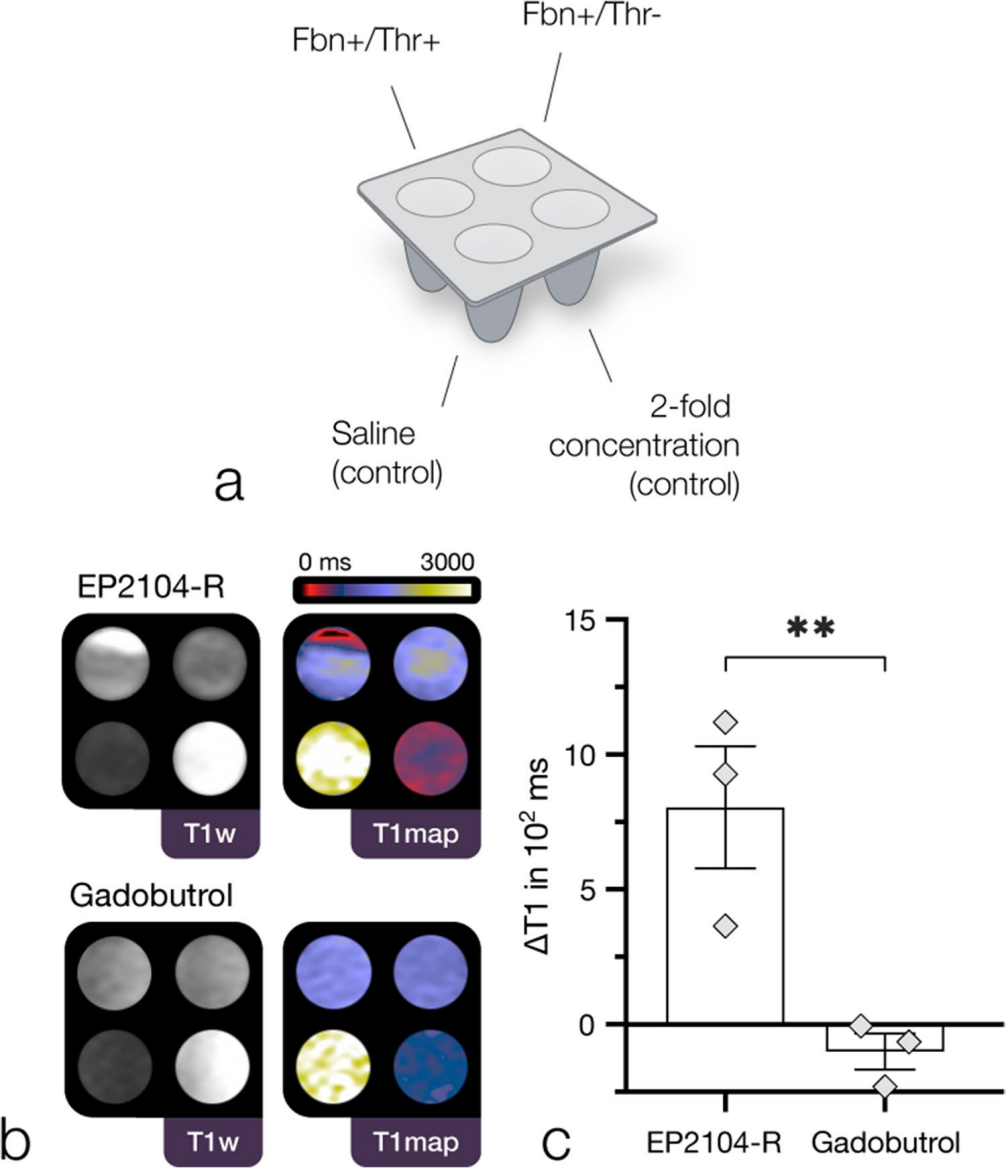
Fibrin binding (in vitro) assay. First panel (**a**) illustrates the experimental setup using 3D-printed multi-well plates (fitted to the phased array coil). Fbn, fibrinogen; Thr, thrombin. Arrangement as shown in panel **b**: top left, Fbn + /Thr + ; top right, Fbn + /Thr-; bottom left, saline (control); bottom right, twofold higher concentration (control). Panel **b** shows a representative binding experiment with EP2104-R (25 µM) and gadobutrol (100 µM): In contrast to EP2104-R, which presented a substantial local decrease in longitudinal relaxation time for wells with fibrinogen and pre-activated thrombin, suggestive for strong binding to the fibrin pellet, gadobutrol exhibits no particular enhancement pattern. Third panel (**c**) shows substantial difference in ∆T1 (determined from respective Fbn + /Thr + and Fbn + /Thr- samples) between EP2104-R and gadobutrol (*p* = 0.009, *n* = 3, independent samples *t*-test, E2.2). *M* ± *SEM*. In the unbound state, EP2104-R exhibits higher relaxivity due to internal motion of the chelate complex. *p*-value < .05 was considered statistically significant. **p-value < 0.01

Fibrin targeting was evaluated in vitro using fitted 3D-printed multi-well plates (see Fig. 2, panel a). Wells (40 µl each) with saline solution (NaCl 0.9%), contrast agent (200 µM gadobutrol or 50 µM EP2104-R) as well as probes containing both human fibrinogen (9.75 mg/ml, Enzo Life Sciences, Inc.) and contrast agent, 100 µM non-specific gadobutrol (Gadovist®, Bayer Pharma AG, Berlin, Germany) or 25 µM EP2104-R (Collagen Medical, LLC, Belmont, MA, USA), including optional treatment with pre-activated human thrombin (1:90, Sigma), were evaluated. Upon brief centrifugation (15 min after treatment with thrombin; 84 × 100 rpm for 5 min) for pellet formation, multi-well plates were examined at room temperature using relaxometry (as described below).

### Experimental autoimmune encephalomyelitis (EAE)

All procedures were approved by the local animal welfare authority (LAGeSo, Berlin, Germany; approval number G 0044/19), and all experiments were performed in accordance with approved guidelines and regulations.

In adoptive-transfer EAE, recipient female SJL/J mice (*n* = 16) at the age of 9–11 weeks receive proteolipid protein (PLP) epitope-primed and epitope-reactivated encephalitogenic T-cells from active EAE donor mice (*n* = 32), which previously developed an acute inflammatory demyelinating disease after immunisation with PLP, as illustrated in Fig. 1, panel e. Additional information is available in Appendix E1 (online).

Mice were monitored daily for body weight and clinical signs according to a 5-point scale as follows: 0 = no disease; 1 = complete tail paralysis; 2 = hindlimb paresis; 3 = hindlimb plegia; 4 = paraplegia and forelimb weakness; and 5 = moribund or death. A humane endpoint was set to an EAE score > 3 in accordance with the approved guidelines. Animals were excluded in the case of non-typical disease course or technical problems. A total of 12 animals were included in this study; 2 animals were killed at the humane endpoint; 2 animals were excluded due to contrast agent extravasation or early remission.

### Immunofluorescence confocal microscopy (IF-CM)

Frozen brain tissue was dissected into 14-μm-thick Sects. (3 consecutive sections). Slides were (co-)stained with FITC-Fibrin(ogen), AF594-(pan-) Laminin, AF594-PLP and AF488-CD45 antibodies. Additional information is available in Appendix E1 (online).

### Laser ablation inductively coupled plasma mass spectrometry (LA-ICP MS)

Using a NWR 213 laser ablation system (New Wave Research, Fremont, CA, USA) and an Agilent 7900 ICP-MS (Agilent Technologies, Japan), tissue was scanned line by line (spot size 60 µm rectangular, line distance 90 µm, 60 µm/s speed, aerosol transport via argon gas flow). ^13^C, ^57^Fe, ^158^Gd and ^139^La isotopes were determined in a semi-quantitative manner through calibration using homogenised tissue standards. An in-house software package was used for colour-coded image reconstruction. Measurements were carried out at Forschungszentrum Jülich (ZEA-3, Jülich, Germany).

### Magnetic resonance imaging (MRI)

MRI was performed on a clinical MAGNETOM (3 T) Biograph mMR (Siemens Healthcare, Erlangen, Germany) using a 4-channel phased array mouse coil (Rapid Biomedical GmbH, Rimpar, Germany). Prior to in vivo MRI, animals were anaesthetised (upon i.p. administration of medetomidine (500 μg/kg), midazolam (5 mg/kg) and fentanyl (50 μg/kg)). Animals were positioned in supine position. Intravenous bolus administration of non-specific gadobutrol (Gadovist®, Bayer Pharma AG, Berlin, Germany) at a clinical dose (100 µmol/kg) and EP2104-R (Collagen Medical, LLC, Belmont, MA, USA), a fibrin-targeted MRI probe, at Gd-equivalent doses (25 µmol/kg) was performed according to total body weight through tail vein (i.v.) injection.

MRI examinations included a pre-injection T2-weighted 3D_TSE sequence (TR/TE = 800/110 ms, resolution = 0.1 × 0.1 × 0.4 mm, FOV = 55 mm, fat-saturated), a pre- and post-injection 3D_MP2RAGE sequence (TR/TE = 5000/6.53 ms, TI1/TI2 = 846/2850, flip angle 1/2 = 4°/5°, resolution = 0.1 × 0.1 × 0.2 mm, FOV = 55 mm) and a pre- and post-injection T1-weighted 3D_VIBE sequence (TR/TE = 13.75/5.68 ms, flip angles = 4°, 8°, 13°, 21°, 34°, resolution = 0.1 × 0.1 × 0.2 mm, FOV = 59 mm). Native and post-contrast T1 mapping was computed inline using data of the 3D_MP2RAGE sequence [19]. Contrast dynamics for the competitive fibrin binding (in vivo) assay were evaluated using iterated sequences.

While clinical MR imaging in MS is usually recommended with a prolonged delay, preferentially 20-min [20] post-administration, allowing for sufficient Gd accumulation of non-specific Gd-based contrast media, fibrin-targeting molecular MRI was performed with a 12-h delay for the purpose of accurate fibrin determination without the impact of non-specific contrast media extravasation. Competitive fibrin binding experiments were comprised of a first scan session pre-/post-contrast, then a 12-h delay upon administration of EP2104-R, a second session prior and after the administration of a lanthanum (La)-labelled analogue (EP2104-La) at tenfold higher dose, which was followed by a second administration of EP2104-R, as shown in Fig. 1, panel d. For the comparative evaluation between targeted and non-targeted contrast media, animals at stable disease peak received a first scan session pre-/post-contrast with gadobutrol, then a 24-h delay to ensure contrast clearance, followed by a second equivalent scan with EP2104-R. Measurements were then performed at a 20-min delay post-contrast phase, although predominant non-specific MR signal response from contrast media extravasation needs to be assumed at this point in time. In addition, a small group of animals (*n* = 3) at disease onset was scanned with EP2104-R only (due to rapid disease course).

### MRI analysis

MRI pre-processing as illustrated in Fig. 1, panel f, included brain extraction using atlasBREX [21], bias-field correction using the N4-method [22] and non-rigid (B-spline) atlas registration using the Advanced Normalization Tools (ANTs) framework [23]. Image analysis was performed using OsiriX MD 11 (Pixmeo, Geneva, Switzerland) in a semi-automated manner (J.L. and M.M., 6 and > 10 years of experience in MR imaging analysis). MRI was correlated with IF-CM and LA-ICP MS using a high-resolution atlas, DSURQE (The Mouse Imaging Centre, Toronto) [24], matched to the respective microanatomy. Native measurements of longitudinal relaxation time were determined using atlas labels (GM/WM/CSF sampled from piriform cortex/internal capsule/lateral ventricle) or semi-automated iso-contouring of EAE lesions presenting a hyperintense correlate in T2-weighted imaging. For the competitive fibrin binding experiment, ROIs were determined on subtraction maps using a region growing algorithm with a fixed threshold value. Periventricular signal enhancement was determined using a standardised ROI in atlas space. Most prominent lesion per subject and matching region in the control group were analysed for association between fibrin(ogen) area and difference in longitudinal relaxation times. Cerebrospinal fluid (CSF) compartments were masked on subtraction maps using a fixed threshold. Semi-automated iso-contouring (minimum 60%) derived regions-of-interest (ROIs) were used for the in vitro fibrin binding assay.

Relative longitudinal relaxation time (rT1) was calculated as ratio of mean longitudinal relaxation time in the region-of-interest (ROI) and the unaffected contralateral background (BG):

1 $$rT1=\frac{{T1}_{mean}^{ROI}}{{T1}_{mean}^{BG}}$$

Signal-to-noise ratio (SNR) for contrast-enhanced T1-weighted imaging was calculated using the following formula (S1, pre-contrast signal; S2, pre-contrast signal; S3, post-contrast signal):

2 $${SNR}_{ROI}=\frac{\sqrt{2 }mean({S3}_{post}-{S2}_{pre})}{std({S1}_{pre}-{S2}_{pre})}$$

### Statistical analysis

Statistics for each experiment are described in both figure legends and text. Error bars represent the S.E.M. *N*-value indicates number of biological replicates. Independent samples *t*-tests or one-tailed paired *t*-tests were used for comparisons between two groups. One-way ANOVA with the Brown-Forsythe test for equal variances and Holm-Šídák’s multiple comparisons test were used for comparing more than two groups. Data distribution was assumed to be normal based on descriptive statistics, the Shapiro-Wilk test and Q-Q plots (available in Appendix E2 (online)). Linear regression was used to model the relationship between variables. *p*-value < 0.05 was considered statistically significant. Each statistical test is reported with exact *p*-value, *n*, *F* and degrees of freedom. Statistical analysis was performed using Prism v.9 (GraphPad Software, San Diego, CA, USA).

## Results

### *Fibrin binding (*in vitro*) assay*

EP2104-R demonstrated high-affinity binding to fibrin in vitro (see Fig. 2, panel b). While there was no particular enhancement pattern for the precursor protein, strong local contrast enhancement was apparent (*M* ± *SD*, 1366 ± 393 ms (fibrinogen) vs. 562 ± 86 ms (fibrin), *p* = 0.04, *n* = 3, *t*(2) = 3.544, one-tailed paired *t*-test, E2.1) when fibrinogen was treated with activated thrombin, resulting in rapid proteolytic conversion to soluble fibrin monomers, which subsequently assemble to fibrin protofibrils and fibres. Correspondingly, there was a substantial difference in longitudinal relaxation time (*M* ± *SD*, 804 ± 393 ms (EP2104-R) vs. − 101 ± 117 ms (gadobutrol), *p* = 0.009, *n* = 3, *t*(4) = 3.824, independent samples *t*-test, E2.2) between gadobutrol, a clinically used (non-targeted) MRI agent, and EP2104-R at equivalent Gd concentrations (see Fig. 2, panel c).

### *Competitive fibrin binding (*in vivo*) assay*

**Fig. 3:**
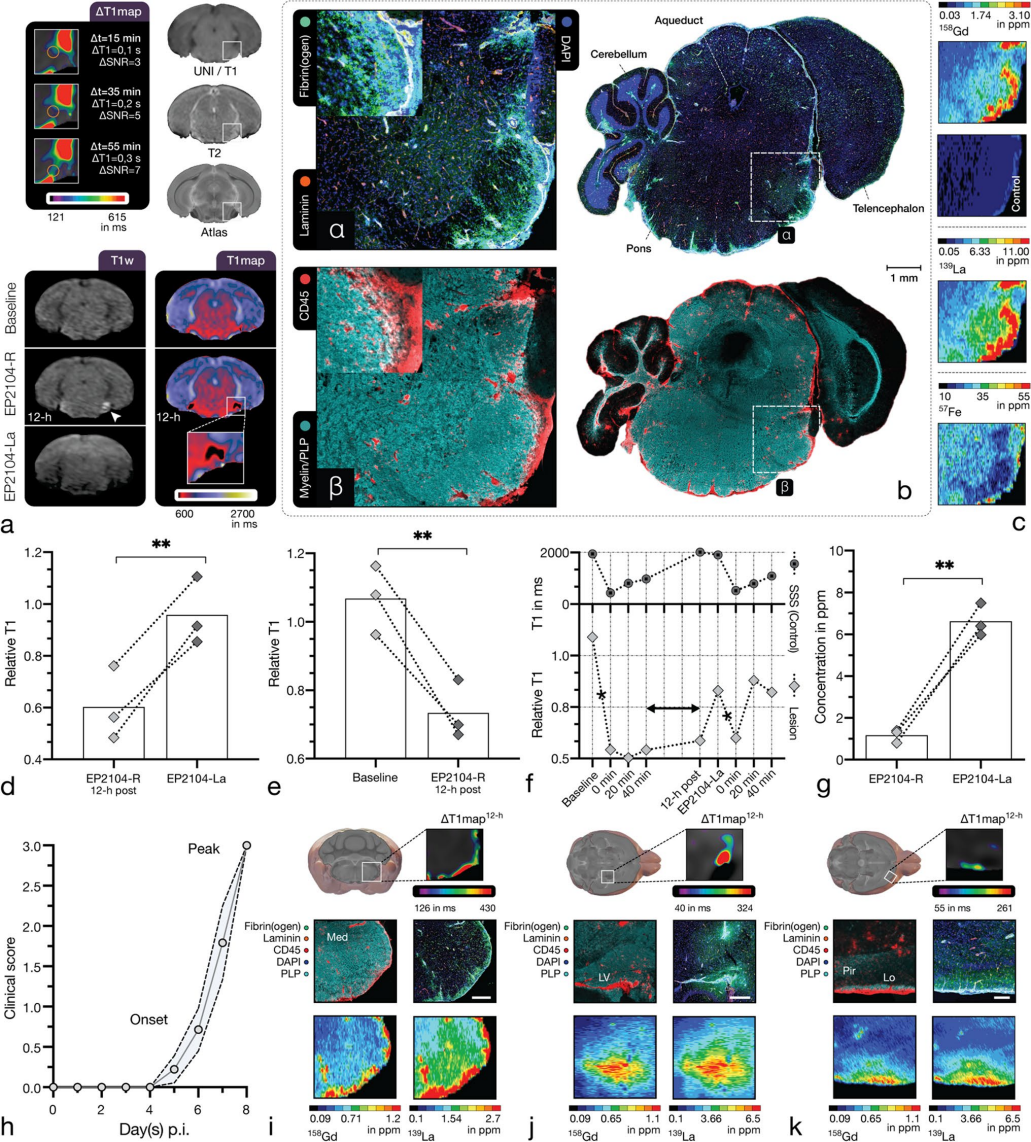
Competitive fibrin binding (in vivo) assay. Competitive binding of EP2104-R to the target protein was evaluated in adoptive-transfer EAE mice at disease peak using a lanthanum-labelled analogue (EP2104-La). Panel **a** shows a representative pontine EAE lesion with prolonged Gd uptake. More than 12-h after the administration of EP2104-R, fibrin-specific contrast enhancement (white arrow) is still apparent, which is displaced with the administration of EP2104-La at tenfold higher dose due to competitive binding. The following panels demonstrate correlated IF-CM (**b**) and LA-ICP MS (**c**). Extensive fibrin(ogen) deposits (**α**) in the brainstem were co-localised with CD45-positive immune cell infiltrates and mild demyelination (**β**). Within EAE lesions, there was a decrease (*p* = .008, *n* = 3, paired one-tailed *t*-test, E2.3) in relative longitudinal relaxation time between baseline and 12-h post-contrast scans (see **e**). When an excess of EP2104-La, an MRI-inactive analogue of EP2104-R, was administered, an increase (*p* = .006, *n* = 3, paired one-tailed *t*-test, E2.4) in relative longitudinal relaxation time was apparent (panel **d**), consistent with displacement of EP-2104-R from its fibrin binding site, as shown in panel **f**. Mean relative longitudinal relaxation time of an EAE lesion is shown over time. T1 values within the superior sagittal sinus (SSS) show venous contrast dynamics for comparison. Asterisk indicates administration of EP2104-R. Correspondingly, LA-ICP MS of tissue slides showed a statistically significant difference (*p* = .003, *n* = 3, paired one-tailed *t*-test, E2.5) between Gd (EP2104-R) and La (EP2104-La) in EAE lesions (see **g**). Panel **h** shows a typical disease course in adoptive-transfer EAE (*n* = 9; pooled from 2 experiments; dotted line, SEM). The following panels (**i**–**k**) show common EAE lesions with corresponding fibrin binding several hours after the administration of EP2104-R. Leptomeningeal inflammation and fibrin binding with extensive myelin damage within the medulla (Med) are shown in panel **i**. Centripetal distribution of fibrin(ogen) and immune cell clustering indicates that inflammation initiates from the meninges (and small perforating venules). Next panel (**j**) shows a prevalent pattern with inflammation and CNS migration through the choroid plexus into periventricular regions (LV, lateral ventricle). Last panel (**k**) illustrates a cortical lesion with subpial and leptomeningeal fibrin deposition with associated immune infiltration and myelin damage affecting the lateral olfactory tract (Lo) and the piriform cortex (Pir). Scale bars, 1 mm (**b**), 500 µm (**i**, **j**), 250 µm (**k**). *p*-value < 0.05 was considered statistically significant. **p-value < .01

Contrast-enhanced MR-neuroimaging was performed in two sessions with a 12-h delay in adoptive-transfer EAE (*n* = 3) at stable disease peak (see Fig. 1). As shown in Fig. 3, EP2104-R demonstrated targeted binding to fibrin deposits in the extracellular matrix (ECM) with corresponding contrast enhancement and decreased longitudinal relaxation time (*M* ± *SD*, 1.07 ± 0.10 (baseline) vs. 0.73 ± 0.09 (EP2104-R), *p* = 0.008, *n* = 3, *t*(2) = 7.969, paired one-tailed *t*-test, E2.3), until several hours after administration (see Fig. 3, panels e and f). Correlated IF-CM showed fibrin(ogen) deposition in the ECM (see Fig. 3, subpanel α) associated with leucocyte infiltration and demyelination (see Fig. 3, subpanel β). When EP2104-La, an analogue where the paramagnetic Gd^3+^ ions were replaced by diamagnetic La^3+^, such that EP2014-La binds fibrin, but does not elicit an MR signal response, was administered at tenfold dose, signal change was reversed and an increase in longitudinal relaxation time (*M* ± *SD*, 0.60 ± 0.14 (EP2104-R) vs. 0.96 ± 0.13 (EP2104-La), *p* = 0.006, *n* = 3, *t*(2) = 8.778, paired one-tailed *t*-test, E2.4) was apparent, suggesting that EP2104-R dissociated from its target due to competitive fibrin binding (see Fig. 3, panel d). When EP2104-R was administered at a second time point (shortly after the administration EP2104-La), relaxation measurements over time imply that a relevant proportion of the early signal change is non-specific, suggesting that accurate fibrin quantification requires equilibrium state (see Fig. 3, panel f). As shown in Fig. 3, panels c and g, LA-ICP MS confirmed the presence of La^3+^ and Gd^3+^ in EAE lesions with corresponding difference between both metals (*M* ± *SD*, 1.17 ± 0.32 ppm (EP2104-R) vs. 6.63 ± 0.79 ppm (EP2104-La), *p* = 0.003, *n* = 3, *t*(2) = 12.85, paired one-tailed *t*-test, E2.5). Representative EAE lesions with leptomeningeal, cortical and periventricular fibrin binding are demonstrated in Fig. 3, panels i–k, presenting correlation between MRI, LA-ICP MS and IF-CM.

### Fibrin deposition in adoptive-transfer EAE

**Fig. 4:**
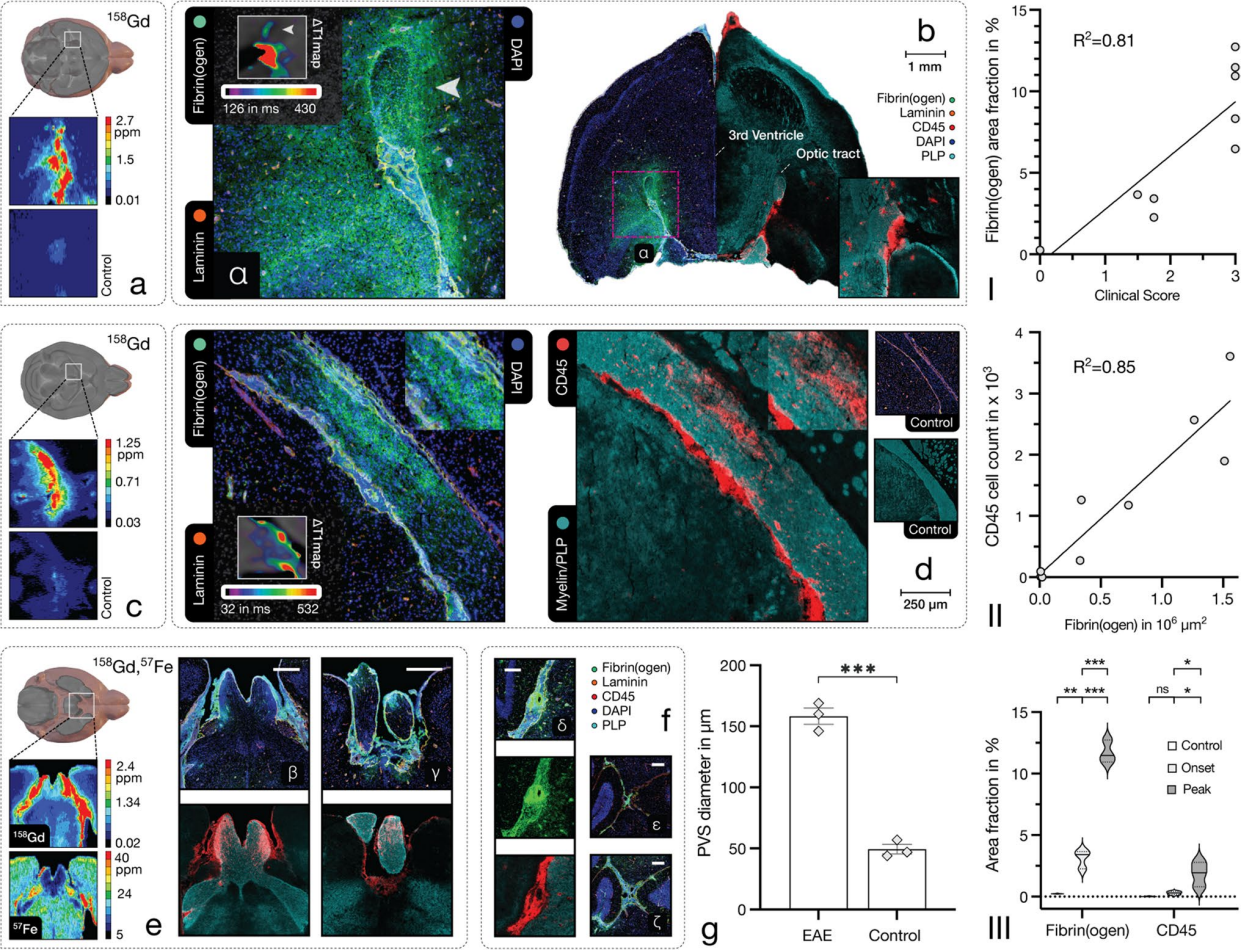
Fibrin(ogen) deposition and fibrin-targeting molecular MRI. Fibrin(ogen) deposition corresponded to clinical disability and disease score (R^2^ = 0.81, *p* < .001, *n* = 11) as well as with immune infiltration (R^2^ = 0.85, *p* < .001, *n* = 9) in EAE, as shown in panel **I** and **II**. Panel **III** shows group differences in fibrin(ogen) and CD45 area fraction between control, disease onset and disease peak (from left to right) samples (*n* = 3, one-way ANOVA with Holm-Šídák’s multiple comparisons test; E2.6, fibrin(ogen), E2.7, CD45) illustrated as a violin plot. The following panels (**a**–**e**) demonstrate molecular imaging of CNS fibrin deposition in early disease stages: Panels **a** and **b** present periventricular inflammation with corresponding decrease in longitudinal relaxation time and interstitial Gd detection in tissue samples. Panel **b** shows matching immunohistopathology (same hemisphere of two adjacent slices) with fibrin(ogen) deposits and immune infiltrates with associated demyelination of the optic tract (see white arrow). Prominent CNS migration through the choroid plexus was apparent in an adjacent slide, demonstrated in the inset. Panels **c** and **d** show inflammation affecting the ventral hippocampal commissure and fimbria with respective decrease in longitudinal relaxation time and Gd detection in LA-ICP MS. Centrifugal fibrin(ogen) deposits, immune cell infiltration and pronounced demyelination adjacent to the lateral ventricle, as shown in panel **d**, suggest leucocyte migration through the choroid plexus. Panel **e** shows extensive inflammation of the optic chiasm (**β**) and optic nerve (**γ**) with associated fibrin(ogen) deposition and Gd detection. Panel **f** demonstrates the enlarged subarachnoid space in EAE: Inflammation within the enlarged subarachnoid space (**δ**) is associated with fibrin(ogen) deposition, which is apparent in MRI with EP2104-R. Subpanels (**ε**, **ζ**) compare the enlarged cerebellar subarachnoid space in EAE (**ζ**) to a control (**ε**). Correspondingly, there was a statistically significant enlargement of the subarachnoid space in EAE compared to healthy controls (*p* < .001, *n* = 3, independent sample one-tailed *t*-test, E2.8), which is shown in panel **g**. *M* ± *SEM*. Scale bars, 1 mm (**b**), 250 µm (**d**), 200 µm (**β**–**δ**), 250 µm (**ε**, **ζ**). *p*-value < 0.05 was considered statistically significant. *p-value < .05, **p-value < .01, ***p-value < .001. ns, non-significant

Fibrin(ogen) deposition progressed with the disease stage (see Fig. 4, panel III) and was associated with progressive leucocyte infiltration and myelin damage. CNS pathology changed from regionally contained at disease onset to more diffuse and extensive inflammation at disease peak with abundant small vessel inflammation and increasing perivascular infiltrates. As shown in Fig. 4, panels I and II, fibrin(ogen) presented a strong association with clinical disability (R^2^= 0.81, *p* < 0.001, *n* = 11) and CD45-positive immune cell infiltration (R^2^ = 0.85, *p* < 0.001, *n* = 9). No fibrin(ogen) deposition or immune cell infiltration was apparent in healthy controls. Leptomeningeal and periventricular inflammation was prominent throughout disease stages. Inflammation in pontine and medullar regions demonstrated centripetal (inward) fibrin(ogen) distribution (see Fig. 3, panels b and i), suggesting that inflammatory activity initiated from the meninges and small perforating vessels. Centrifugal (outward) fibrin(ogen) distribution was typically associated with choroid plexus, perivenular and subarachnoidal pathology (see Fig. 4, panels a–d). Inflammation of the optic chiasm and optic nerve was apparent and showed corresponding fibrin(ogen) deposition, as illustrated in Fig. 4, panel e. Cortical lesions presented association with the pial surface and (sub-)cortical perforating venules (see Fig. 3, panel k).

The subarachnoid space was enlarged at disease peak due to severe immune cell trafficking (*M* ± *SD*, 158 ± 0.12 µm (EAE) vs. 49 ± 7 µm (control), *p* < 0.001, *n* = 3, *t*(4) = 14.05, independent sample one-tailed *t*-test, E2.8), as shown in Fig. 4, panel g, and showed small surrounding clusters of immune cells migrating into brain parenchyma. Laminin-immunostaining depicted basement membranes for further differentiation between lumen, perivascular space and subarachnoidal space. As demonstrated in Fig. 4, panel f, extensive fibrin(ogen) deposition was present in the subarachnoid and perivascular compartment, which was apparent in imaging with EP2104-R, and coincided with strongly dysregulated immune cell trafficking.

**Fig. 5:**
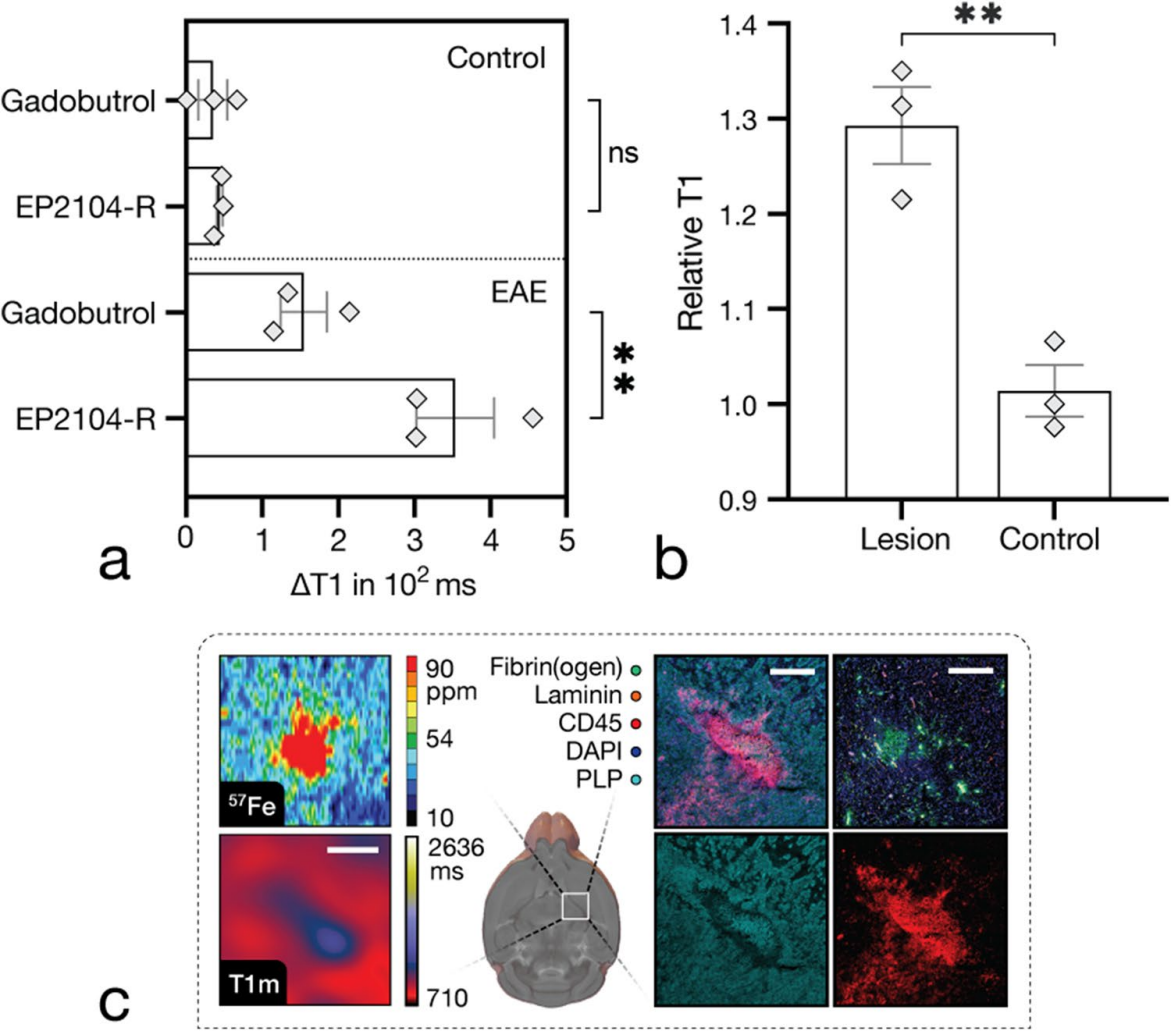
Comparison between targeted and conventional MRI probes. First panel (**a**) demonstrates a comparison between EP2104-R and gadobutrol (as a clinically used non-targeted MRI contrast agent) in periventricular inflammation. EP2104-R presented greater reduction in longitudinal relaxation time (*p* = .006, *n* = 3, paired one-tailed *t*-test, E2.9) compared to gadobutrol at Gd ion-matched dose (20-min delay post-administration). *M* ± *SEM*. Panel **b** shows increased longitudinal relaxation times (*p* = .005, *n* = 3, independent sample two-tailed *t*-test, E2.11) in large EAE lesions compared to controls. *M* ± *SEM*. Last panel (**c**) demonstrates a representative late active EAE plaque in the internal capsule with increased longitudinal relaxation time. LA-ICP MS revealed an abundance of iron and corresponding immunohistopathology presented a large immune cell conglomerate with extensive myelin damage. Scale bars, 500 µm. *p*-value < 0.05 was considered statistically significant. **p-value < .01. ns, non-significant

As shown in Fig. 5, panel a, EP2104-R showed greater reduction of longitudinal relaxation (*M* ± *SD*, 354 ± 89 (EP2104-R) vs. 155 ± 53 (gadobutrol), *p* = 0.006, *n* = 3, *t*(2) = 9.199, paired one-tailed *t*-test, E2.9) as well as higher SNR (*M* ± *SD*, 6.6 ± 1 (EP2104-R) vs. 2.7 ± 0.4 (gadobutrol), *p* = 0.004, *n* = 3, *t*(2) = 10.73, paired one-tailed *t*-test, E2.10) compared to non-specific gadobutrol (at Gd-equivalent dose), when imaged with a 20-min delay post-administration. Difference in longitudinal relaxation time of EP2104-R determined at that time showed an association with corresponding fibrin(ogen) deposition (R^2^ = 0.86, *p* < 0.001, *n* = 9); however, measurements at this point still reflect a mixed component with non-specific signal.

In native T1 mapping, an increased longitudinal relaxation time (*M* ± *SD*, 1.29 ± 0.07 (lesion) vs. 1.0 ± 0.05 (control), *p* = 0.005, *n* = 3, *t*(4) = 5.749, independent sample two-tailed *t*-test, E2.11) was measured in acute EAE lesions compared to controls (see Fig. 5, panel b). Longitudinal relaxation times for GM/WM/CSF were (*n* = 3, *M* ± *SD*) 1311 ± 34/882 ± 9/3281 ± 664 ms in healthy animals.

## Discussion

For the first time (to our knowledge), we demonstrated that CNS fibrin deposition can be determined using molecular MRI, which provides a non-invasive imaging biomarker for inflammatory CNS pathology and barrier impairment. We showed that EP2104-R, a peptide-conjugated Gd-based MRI probe, binds to fibrin deposits with high affinity and specificity. Signal enhancement was apparent in EAE lesions even several hours after administration of EP2104-R due to targeted binding. When EP2104-La, a MRI-silent analogue, was administered at high dose, EP2104-R was displaced from its target due to competitive binding. At the equilibrium state of EP2104-R, fibrin deposition may be determined as a quantitative non-invasive imaging biomarker that is strongly associated with immune infiltration, which is directly linked to myelin damage and axonal injury [5, 25]. We demonstrated that fibrin(ogen) deposition progressed with disease stage and corresponded to clinical disability. Fibrin-targeting MRI also revealed extensive deposits in the enlarged perivascular and subarachnoid compartment, which could be visualised and coincided with abnormal cell trafficking.

EP2104-R exhibits high relaxivity as a result of accumulation through specific binding in addition to its internal rotation mechanism [14, 26]. The differences in longitudinal relaxation time and SNR were substantially higher at Gd-equivalent dose than with a non-targeted MRI contrast agent. Lesions with decreased barrier permeability at the time of imaging may be underestimated with clinically used (non-targeted) MRI contrast agents. On the opposite, EP2104-R binds fibrin and gradually accumulates in lesions, increasing MR signal enhancement, which improves the detection of enhancing lesions and, therefore, has the potential to improve the clinical diagnostic capacity, particularly with regard to cortical lesions, which were shown to be main contributors to disability in MS [27], but were reported to be underestimated using clinical MRI [28]. Subpial lesions are characteristic and prevalent cortical pathology in MS [9] and are commonly associated with leptomeningeal inflammation [29]. Our findings suggest that molecular imaging of CNS fibrin deposition might aid the clinical detection of leptomeningeal and cortical inflammation—clinically relevant to MS because of an association with secondary progression, disability and cognitive impairment [30]—due to substantially increased SNR and the possibility of imaging with prolonged delays that exclude the presence of non-specific enhancement in meningeal blood vessels or leakage of contrast media into the surrounding CSF, which may further mask inflammatory foci.

We also observed fibrin(ogen) deposition in the visual pathway, which is an interesting target for the assessment of acute inflammatory events in clinically isolated syndrome (CIS) where optic neuritis is frequently the first clinical manifestation of MS [9, 31]. Further research should evaluate the prognostic value of CNS fibrin deposition for the conversion from CIS to manifest MS.

Moreover, there is potential for dosage reduction—a relevant topic in light of more recent reports of Gd deposition upon serial contrast-enhanced MRI [32]. With regard to reports about increased sensitivity for enhancing lesions with double- and triple-dosing [33, 34], which is not appropriate according to the 2017 revision of the McDonald criteria [28], molecular MRI probes, such as EP2104-R, which provide high relaxivity and specific target binding, appear to be relevant alternative solutions. Furthermore, a Mn-based alternative to EP2104-R was developed [15], which would eliminate the risk of Gd deposition entirely.

While advanced neuroimaging methods [35, 36], such as diffusion tensor imaging (DTI) or magnetisation transfer ratio (MTR), were shown to complement structural MRI, their availability is commonly limited to research environments due to extensive data and hardware requirements with time-consuming measurements and image pre-processing. On the other hand, we demonstrated that molecular MRI is a highly translational approach by using a clinical high-field MRI scanner and the generally available MP2RAGE sequence [19] for fast and robust T1 relaxometry at low transmit-field inhomogeneity. In the current study, acute inflammatory lesions demonstrated an increase in longitudinal relaxation time, suggestive for myelin damage and/or vasogenic edema. When T1 mapping is performed prior to and after the administration of contrast agent, subtraction (ΔT1) maps reflect the change in longitudinal relaxation time primarily induced by the MRI probe, which is a function of local concentration.

### Fibrin deposition as a disease biomarker

Fibrin presents a superior cumulative measure for barrier impairment compared to the extravasation measurement of small externally applied agents, such as non-targeted Gd-based contrast agents or fluorescent probes, as fibrin polymers remain in the interstitial space as an insoluble mesh network until proteolytic degradation to fibrin-degradation products (FDP) [1]. However, more importantly, fibrin deposition is a promising biomarker for CNS pathology due to its pathophysiological implications in neurological diseases [1]. There is a growing body of evidence that shows that fibrin deposition plays a role in various inflammatory and neurodegenerative CNS diseases [1], such as MS where extensive fibrin deposits were demonstrated in acute/chronic lesions [37, 38] and persistent dysregulation of the fibrinolytic pathway was reported [39]. Fibrin was also found in the cortex of patients with MS, where its distribution correlated with neuronal loss [40]. CNS fibrin was shown to contribute to the EAE disease course in pre-clinical studies, where disease severity was alleviated using experimental therapeutic agents that block the interaction of fibrin with the CD11b integrin receptor on microglia and macrophages [41] or inhibit the conversion of fibrinogen to fibrin using a thrombin inhibitor [5].

### Limitations

Despite certain limitations of the EAE disease model (see [42] for further details), it is still highly relevant to date, due to its significant contributions to the investigation of inflammatory and demyelinating CNS disorders. Although fibrin-targeting molecular MRI was evaluated and demonstrated in a murine model, CNS fibrin deposition is neither exclusive to EAE nor MS, but presents a critical and fundamental event in the pathophysiology of several CNS disorders [1] with variable degrees of barrier impairment, ranging from autoimmune encephalitis, vasculitis to CNS infections, suggesting that data from this model might be relevant to an even broader spectrum of neurological conditions. Although transitory disruption of the barrier function may occur in several neurological diseases, only a subset of CNS disorders with profound dysregulation of the immune system present a sustained transformation of the neurovascular interface to a proinflammatory milieu, such as in MS (as discussed above) or Alzheimer’s disease, where CNS fibrin(ogen) was also shown to play a role in its pathophysiology [1, 43–45]. Hence, CNS fibrin deposition as an imaging biomarker is not specific to a single neurological disease entity, but it yields high sensitivity for the inflammatory component of several CNS pathologies and fibrin-targeting molecular MRI may improve the detection of subtle inflammatory foci, such as in cortical inflammation in MS.

Another limitation is that the administration of contrast media could not be randomised due to prolonged fibrin binding. Although gadobutrol was administered 24-h prior to EP2104-R, an underestimation at clinically and radiologically stable disease peak appears unlikely.

Although molecular imaging using PET can potentially provide biologically relevant information without barrier dysfunction, inherently low spatial resolution makes the detection of most MS or EAE lesions difficult. While increased barrier permeability is a prerequisite for fibrin-targeting molecular MRI, literature suggests that dysfunction of the BBB occurs at an early stage in MS and precedes damage [1].

In conclusion, we demonstrated that CNS fibrin deposition can be determined using molecular MRI and proposed fibrin deposition as an imaging biomarker for CNS pathology, which is sensitive to disease activity, and acts as a surrogate for neuroinflammation and barrier impairment.

## Supplementary Information

Below is the link to the electronic supplementary material.

Supplementary file1 (PDF 68.4 KB)

Supplementary file2 (PDF 673 KB)

## Data Availability

All data generated or analysed during this study are included in this published article (and its supplementary information files).

## Abbreviations

CNS: Central nervous system
Gd: Gadolinium
La: Lanthanum
EAE: Experimental autoimmune encephalomyelitis
