# Supplementary material for: Fibrin-targeting molecular MRI in inflammatory CNS disorders
Source: Eur J Nucl Med Mol Imaging. 2022 May 4;49(11):3692–704. doi: 10.1007/s00259-022-05807-8 (PMC9399196; doi:10.1007/s00259-022-05807-8)
Supplement: Supplementary file 1 — Supplementary file1 (PDF 68.4 KB) [file 259_2022_5807_MOESM1_ESM.pdf]

Appendix E1

Experimental autoimmune encephalomyelitis (EAE)

Female SJL/J mice (Janvier Labs, St Berthevin Cedex, France) at the age of 9 - 11 weeks were housed under specific-pathogen-free (SPF) conditions with a 12 hours light/dark photoperiod cycle and had unrestricted access to high caloric nutrition and water. Health and neurological status were closely monitored throughout the study.

Adoptive-transfer EAE isolates the effector phase of the demyelinating disease starting with CNS migration of encephalitogenic T-cells mediated through pro-inflammatory cytokines and chemokines. With dysregulation and impairment of the BBB, the passage for further peripheral leucocyte migration into the CNS is paved. Extravasated fibrin(ogen) further promotes and potentiates the inflammatory response (as discussed). Activated peripheral and resident immune cells then induce CNS injury through phagocytosis and the release of inflammatory agents, such as cytokines or reactive oxygen species.

Naïve SJL/J mice (n = 32) were immunised with an emulsion containing 250 µg PLP (murine proteolipid peptide p139-151, Pepceuticals, Leicester, United Kingdom) in equal volumes of PBS and Complete Freund's Adjuvant (CFA, Thermo Fischer Scientific, USA) and 4 mg/ml inactive Mycobacterium tuberculosis H37Ra (Difco Laboratories, Detroit, MI, USA). Pertussis toxin (250 µg, List Biological Laboratories, Inc., Campbell, CA, USA) was injected i.p. on the day of immunisation. Starting with immunisation (designated as day 0), mice were monitored daily for body weight and clinical signs according to a 5-point scale as follows: 0 = no disease; 1 = complete tail paralysis; 2 = hindlimb paresis; 3 = hindlimb plegia; 4 = paraplegia and forelimb weakness; and 5 = moribund or death. A humane endpoint was set to an EAE score > 3 in accordance to the approved guidelines. At disease peak, cells were extracted from draining lymph nodes and restimulated with 12.5 µg PLP/ml in cell culture medium (RPMI 1640 supplemented with 2 mM L-glutamine, 100 U/ml penicillin, 100 µg/ml streptomycin and 10 % fetal calf serum) for 72-h at 37°C. After intraperitoneal (i.p.) injection of (approx. 30 $x10^6$) PLP-reactive T-cells into naïve syngeneic recipients (n = 16), mice developed adoptive-transfer EAE, clinically apparent as ascending paralysis with rapid onset.

Mice were killed in deep anaesthesia (upon i.p. administration of medetomidine (500 µg/kg), midazolam (5 mg/kg) und fentanyl (50 µg/kg)) through cardiac perfusion with PBS. Brains (without spinal cord) were immediately removed, bisected at the junction from mid-

brain to cerebellum, snap-frozen in Tissue-Tek® OCT compound (Sakura Finetek Germany GmbH, Staufen, Germany) and stored at -80 °C.

Immunofluorescence Confocal Microscopy (IF-CM)

Slides were (co-)stained with Goat anti-mouse FITC-Fibrin(ogen) (1:200, GAM/Fbg/7S, Nordic MUbio, Susteren, Netherlands), AF594-(pan-)Laminin (1:200, NB300-144AF594, Bio-Techne Ltd., Minneapolis, MN, USA), AF594-PLP (1:100, bs-11093R-A594, Bioss Inc., Woburn, MA, USA) and AF488-CD45 (1:100, NB100-77417AF488, Bio-Techne Ltd., Minneapolis, MN, USA) antibodies overnight upon blocking with a non-protein-based blocking solution. Sections were rinsed, coated with ROTI Mount FluorCare DAPI (Carl Roth GmbH + Co. KG, Germany) and scanned with a ZEISS Axio Observer Z1 microscope with Apotome (Carl Zeiss MicroImaging GmbH, Jena, Germany). Confocal microscopy was (semi-) automatically quantified as area and area fraction or cell count using a static threshold-based approach. Area fraction was measured in representative whole brain slides and respectively matching control slides. Area and cell count were determined in the most prominent EAE lesion per subject and respectively matching control slides. Data collection and analyses was performed using ImageJ 2.1.0/1.53c (NIH), Python 3 and Zeiss ZEN Software.
