# Supplementary material for: Fibrin-targeting molecular MRI in inflammatory CNS disorders
Source: Eur J Nucl Med Mol Imaging. 2022 May 4;49(11):3692–704. doi: 10.1007/s00259-022-05807-8 (PMC9399196; doi:10.1007/s00259-022-05807-8)
Supplement: Supplementary file 2 — Supplementary file2 (PDF 673 KB) [file 259_2022_5807_MOESM2_ESM.pdf]

Appendix E2

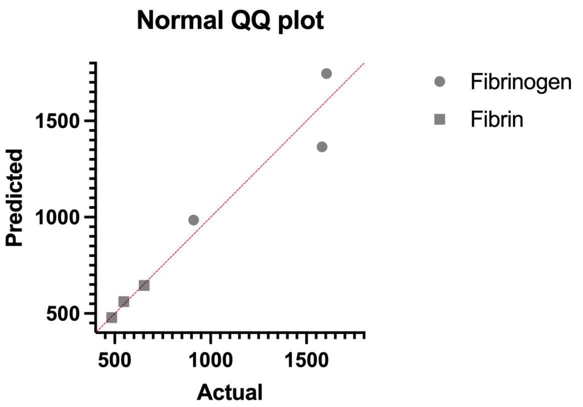

E2.1. Q-Q plot

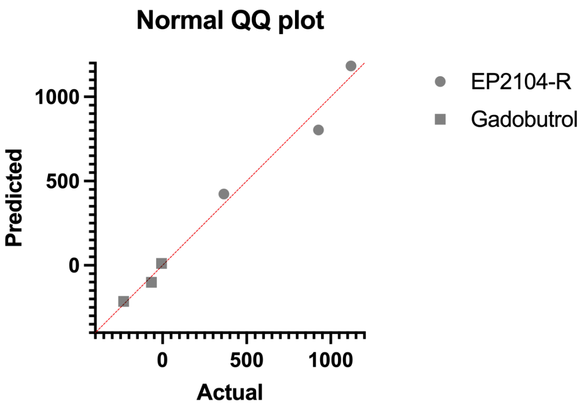

E2.2. Q-Q plot

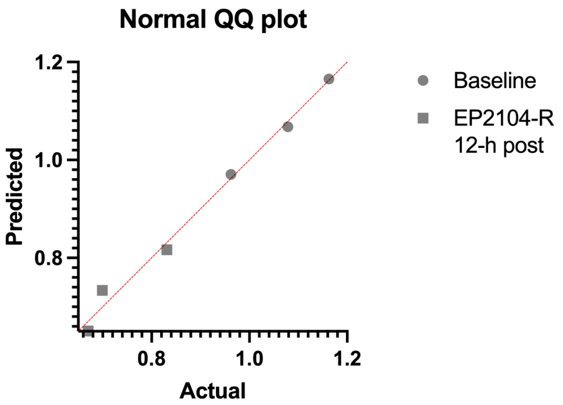

E2.3. Q-Q plot

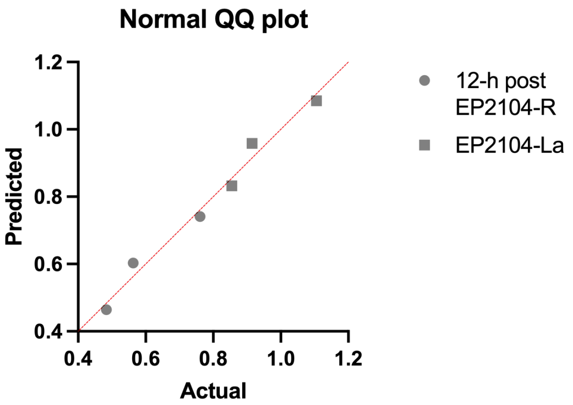

E2.4. Q-Q plot

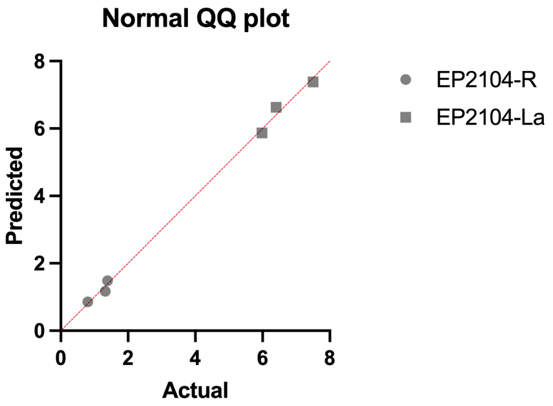

E2.5. Q-Q plot

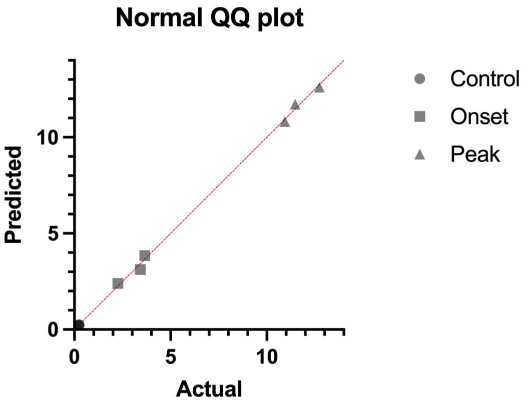

E2.6. Q-Q plot

Appendix E2

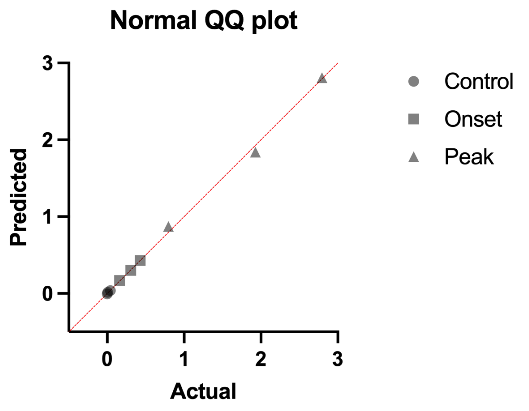

E2.7. Q-Q plot

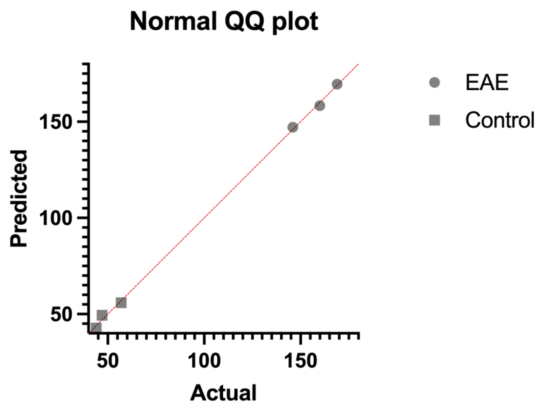

E2.8. Q-Q plot

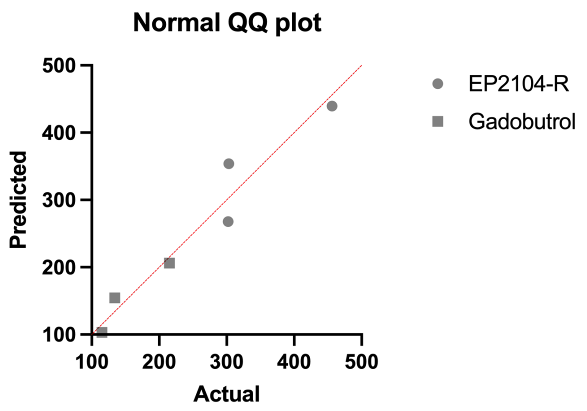

E2.9. Q-Q plot

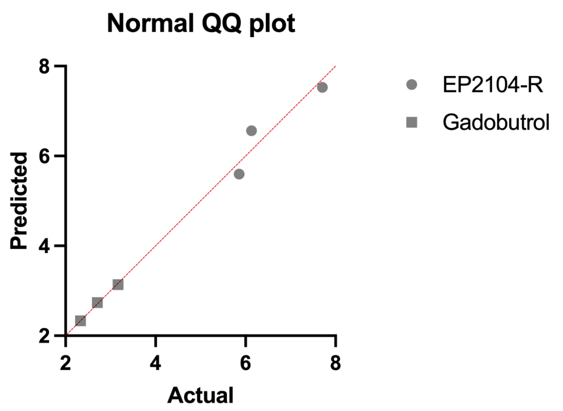

E2.10. Q-Q plot

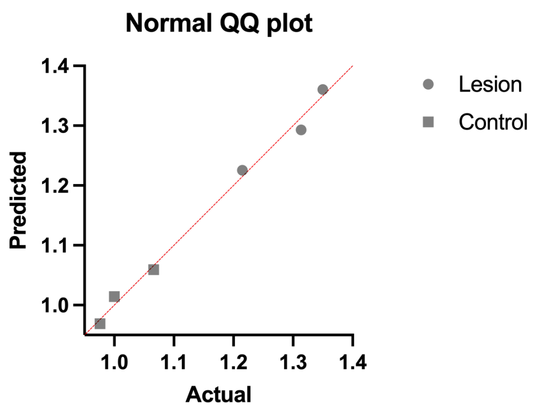

E2.11. Q-Q plot
